# Analytical Expressions for Numerical Characterization of Semiconductors per Comparison with Luminescence

**DOI:** 10.3390/ma11010002

**Published:** 2017-12-21

**Authors:** Mauro F. Pereira

**Affiliations:** Department of Condensed Matter Theory, Institute of Physics CAS, Na Slovance 1999/2, 182 21 Prague, Czech Republic; pereira@fzu.cz; Tel.: +420-266-052-153

**Keywords:** luminescence, absorption, dilute semiconductors, many body effects, TERA-MIR radiation

## Abstract

Luminescence is one of the most important characterisation tools of semiconductor materials and devices. Recently, a very efficient analytical set of equations has been applied to explain optical properties of dilute semiconductor materials, with an emphasis on the evolution of peak luminescence gain with temperature and its relation to sample quality. This paper summarizes important steps of the derivation of these expressions that have not been presented before and delivers a theoretical framework that can used to apply exactly solvable Hamiltonians for realistic studies of luminescence in various systems.

## 1. Introduction

Materials development requires characterization techniques. Among them, photoluminescence, or in more general terms, radiation emission due to different excitation mechanisms [1], is a very powerful tool to study semiconductor materials and map specific characteristics of new devices [2,3] for applications from the THz-Mid Infrared (TERA-MIR) to ultraviolet ranges [4,5,6]. A one-to-one correspondence between measured spectra and fundamental materials properties requires a clear theoretical model, ideally easy to understand and to programme, but at the same time with microscopic information for conclusive interpretation and as free as possible from phenomenological parameters.

A recent theoretical effort led to the development of analytical solutions for the interband polarization, which plays the selfenergy role in the Dyson equation for the Photon Green’s functions [7], which have been applied them to study photoluminescence of Coulomb-correlated semiconductor materials. The accuracy of the resulting easily programmable solutions has been demonstrated by consistently explaining the low temperature s-shape of the luminescence peak of dilute semiconductors, such as ternary GaAsSb, InAsN, and quaternary InAs(N,Sb) [7,8,9]. The interplay of homogeneous versus inhomogeneous broadening at low and high temperatures are described, together with the relevance of many body effects, which are in very good agreement with experiments [10,11,12,13]. A similar set of equations was also used to study nonlinearities in GaAs–AlGasAs and GaAsN–AlGaAs superlattices [14,15,16]. The superlattice case is particularly noteworthy for room temperature GHz nonlinear multiplication into the THz range [17,18]. This paper has two objectives: to show hitherto unpublished details of the mathematical steps that lead to the equations used in Refs. [7,8,9,14,15,16], and to draw a bridge between the luminescence and nonlinear absorption calculations in superlattices. It is organized as follows: the main steps involving manipulations of hypergeometric functions that characterize the solution of the Hulthén potential problem are delivered. Next, a direct connection between luminescence and absorption equations is given together with a connection with generalized semiconductor Bloch equations, with potential for the study of polaritons in superlattices within a dielectric approach such as that used to investigate valence band THz polaritons and antipolaritons [6]. A brief conclusion follows.

## 2. Mathematical Model

In order to make this paper self-contained, some of the steps shown in Ref. [7] are followed to guide the presentation towards the more complete derivation presented here.

### 2.1. Integro-Differential Equation for the Power Spectrum

Luminescence, or equivalently the optical power density spectrum I(ω), is described quantum mechanically by the Poynting vector, which can be directly related to the transverse polarization function P, which is the selfenergy in the Dyson equations for the transverse photon Green’s function components in the Keldysh formalism [7,19,20]. All the quantities presented in this paper are considered in frequency space, i.e., evaluated at steady state.

(1) $$I\left(\omega \right)=\left(\hbar \omega^{2}/4\pi^{2}c\right)\;iP^{<}\left(\omega \right)$$

The free photon Green’s function represents the photons propagating without any interaction with the medium. When carriers are injected the transverse polarization function P, which is the selfenergy in photon Green’s function Dyson equation, determines how the excited medium modifies the photon propagation. The lesser Keldysh component $P^{<}$ is proportional to the carriers recombination rate and yields the number of emitted photons per unit area. It thus governs the power emission spectrum, as seen in Equation (1). The imaginary and real parts of $P^{r}$ are, respectively, proportional to absorption and gain and refractive index changes, since the dielectric function of the medium reads $\epsilon \left(\omega \right)=1-\frac{c^{2}}{\omega^{2}}P^{r}\left(\omega \right)$ as shown in Ref. [19]. The starting points for the results derived here are the equations in Refs. [7,19,20].

(2) $$P^{r/<}\left(\omega \right)=\frac{4\pi e^{2}{\left|\Pi \right|}^{2}}{c^{2}\Omega }\underset{\vec{k}}{\sum }P^{r/<(\vec{k},\omega )}.$$

Here, e,c,Ω, and Π denote, respectively, the electron charge, the speed of light, the sample volume and the velocity matrix element, which is the expectation value of the velocity operator, i.e., the momentum operator divided by the electron mass. It stems directly from the fact that current is charge times velocity. The formal definition of the transverse polarization function selfenergy in terms of functional derivatives is $P=-\frac{4\pi }{c}\frac{\delta J}{\delta A}$, where J and A are, respectively, expectation values of the induced current and vector potential operators. The full expression involves labels along the Keldysh contour and is tensorial. A complete discussion is beyond the scope of this paper. For details see Refs. [19,20].

The crystal momentum $\vec{k}$ is a consequence of Fourier transforming from real space. Likewise, *ω* and thus the photon energy ℏω stem from a corresponding Fourier transformation from time to the frequency domain. The matrix element satisfies the integro-differential equation [20],

(3) $$P^{r}(\vec{k},\omega )={P_{0}}^{r}(\vec{k},\omega )-\underset{\vec{k}}{\sum }{P_{0}}^{r}(\vec{k},\omega )W_{\vec{k}-\vec{k^{\prime }}}P^{r}(\vec{k}\prime ,\omega )$$

where W is the screened Hulthén potential [21,22,23,24]. Furthermore,

(4) $$2Im\{{P_{0}}^{r}(\vec{k},\omega )\}=\int \frac{d\omega^{\prime }}{2\pi }\hat{G_{e}}(\vec{k},\omega^{\prime })\hat{G_{h}}(\vec{k},\omega -\omega^{\prime })\{1-f_{e}(\omega^{\prime })-f_{h}(\omega -\omega^{\prime })\}$$

Electrons or holes are labelled, respectively, by λ={e,h}, the renormalized energies $e_{\lambda }\left(\vec{k}\right)$, and dephasing $\Gamma_{\lambda }$ are calculated from the real and imaginary parts of the selfenergy in the Dyson equation for the retarded carriers Green’s functions. This paper focuses on quasi-equilibrium luminescence and on three dimensional (bulk semiconductors) with one conduction and one valence band.

Under these conditions, $f_{\lambda }$ denotes a Fermi function characterized by a chemical potential $\mu_{\lambda }$ and the spectral function in Equation (4) for each particle, derived from components of the carriers’ Green’s function in the Keldysh formalism, reads

(5) $${\hat{G}}_{\lambda }\left(\vec{k},\omega \right)=\frac{2\Gamma_{\lambda }}{{\left(\hbar \omega -e_{\lambda }\left(\vec{k}\right)\right)}^{2}+\Gamma_{\lambda }^{2}}.$$

The next step is to re-write the last term in Equation (4) by means of the identity

(6) $$1-f_{e}\left(\omega^{\prime }\right)-f_{h}\left(\omega -\omega^{\prime }\right)=\left\{\left[1-f_{e}\left(\omega^{\prime }\right)\right]\left[1-f_{h}\left(\omega -\omega^{\prime }\right)\right]+f_{e}\left(\omega^{\prime }\right)f_{h}\left(\omega -\omega^{\prime }\right)\right\}\tanh\left[\left(\hbar \omega -\mu \right)/\left(2K_{B}T\right)\right]$$

and to approximate this factor by $1-f_{e}\left(\omega^{\prime }\right)-f_{h}\left(\omega -\omega^{\prime }\right)\;\approx \;\tanh\left[\frac{\left(\hbar \omega -\mu \right)}{2K_{B}T}\right]$, where $\mu =\mu_{e}+\mu_{h}$ is the total chemical potential, where *T* is the temperature in Kelvins and $K_{B}$ is the Boltzmann constant. Different versions of this approximation has been used before in phenomenological approaches for absorption Refs. [21,22,23] and delivered good agreement with experiments (see details and further references in Ref. [23]). Within the Keldysh Green’s functions, context, a detailed derivation of its application is given in Ref. [20]. The fully numerical solutions of the equations that use this version of the approximation have given very good agreement with both single beam and pump-probe luminescence [20,25]. Its usefulness has been further confirmed recently by the good agreement between the analytical solutions shown here and the experimental luminescence of dilute semiconductors [7,8,9,14].

**Figure 1:**
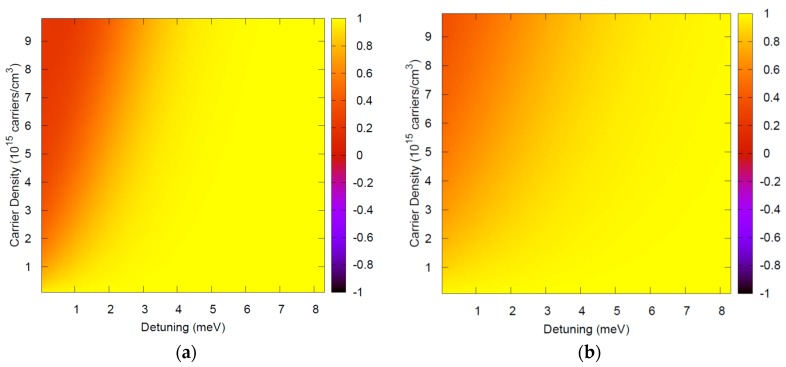
$FF\left(\omega ,\omega^{\prime }\right)$, the term in curly braces in Equation (6) which has been approximated by $FF\left(\omega ,\omega^{\prime }\right)\approx 1$, evaluated at $\hbar \omega =E_{g}$ for bulk GaAs at low temperatures, where the dephasing is typically small $\Gamma_{\lambda }$. In this case, only a small range of detunings ω−ω′ contribute to the integral in Equation (4). Thus the range chosen in the *x*-axis, from zero to approximately twice the exciton binding energy ($2e_{0}$), is even larger than necessary. (**a**) *T* = 10 K; (**b**) *T* = 20 K.

**Figure 2:**
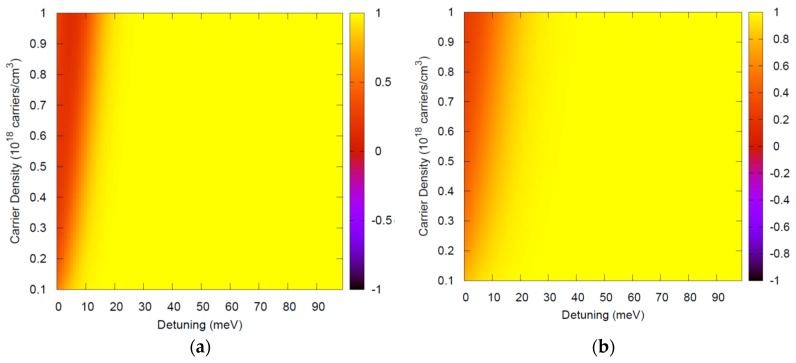
$FF\left(\omega ,\omega^{\prime }\right)$, the term in curly braces in Equation (6) which has been approximated by $FF\left(\omega ,\omega^{\prime }\right)\approx 1$, evaluated at $\hbar \omega =E_{g}$ for bulk GaAs at higher temperatures and high densities, where the dephasing $\Gamma_{\lambda }$ is larger than in the low temperature case. In this case a wider range of detunings ω−ω′ contribute to the integral in Equation (4). Thus, the range chosen in the *x*-axis is even larger than necessary. (**a**) *T* = 150 K, (**b**) *T* = 300 K.

Figure 1 and Figure 2 depict typical ranges of parameters, showing that $FF\left(\omega ,\omega^{\prime }\right)=\left\{\left[1-f_{e}\left(\omega^{\prime }\right)\right]\left[1-f_{h}\left(\omega -\omega^{\prime }\right)\right]+f_{e}\left(\omega^{\prime }\right)f_{h}\left(\omega -\omega^{\prime }\right)\right\}\approx 1$ is an excellent approximation. Note that this theory is applied for photon energies around the semiconductor bandgap and thus in Figure 1 and Figure 2, $\hbar \omega =E_{g}$.

Low temperature luminescence is typically performed with a small density of injected carriers. Very good agreement of this theory with results from different experimental teams for a variety of materials has been obtained with carrier densities around 10^15^ carriers/cm^3^ [7,8,9], further justifying the range of densities in the *y*-axis. The theory has also been used for high temperatures and high densities to investigate optical nonlinearites [14,15,16], and this range is illustrated in Figure 2.

Relevant dephasing mechanisms such as electron-electron, electron-phonon and electron-impurity scattering can be added to the selfenergy [17,18], and the resulting $\Gamma_{\lambda }$ is frequency and momentum dependent. However, in what follows, it is replaced by averaged values, leading to a simple approximation for $Im\{{P_{0}}^{r}(k,\omega )\}$ consistent with the Ansatz solution,

(7) $${P_{0}}^{r}(\vec{k},\omega )\equiv \frac{\vartheta }{\hbar \omega -\Delta e(\vec{k})+i\Gamma }$$

where $\Gamma =\Gamma_{e}+\Gamma_{h}$ and ϑ≡tanh[β(ℏω−μ)/2]. In 3D, the material resonance energy is: $\Delta e\left(\vec{k}\right)=\frac{\hbar^{2}{\left|\vec{k}\right|}^{2}}{2m_{r}}+E_{g}$, where 1m*=1me+1mh. The bandgap $E_{g}$ is given by the sum of the fundamental band gap $E_{g}^{0}$, and a many body renormalisation term $\Delta E_{g}$ where $m_{e},\;m_{h}$ denote, respectively the electron and hole effective masses. The equation for $P^{r}(\vec{k},\omega )$ simplifies to:

(8) $$(\hbar \omega -\Delta e(\vec{k})+i\Gamma )P^{r}(\vec{k},\omega )+\vartheta \underset{\vec{k}\prime }{\sum }W_{\vec{k}-\vec{k^{\prime }}}P^{r}(\vec{k^{\prime }},\omega )=\vartheta$$

The total dephasing Γ will determine the luminescence linewidth. Thus, it can be treated as a phenomenological parameter used to interpret data, and at the same time estimate the strength of the scattering and dephasing processes [7,8,9] by comparison of adjusted data with microscopic calculations derived from the relevant selfenergies [17,18]. At this point, the Kubo–Martin–Schwinger (KMS) relation under the form derived in Ref. [20] can be applied to Equation (8),

(9) $$P^{<}\left(\omega \right)=\frac{-2iIm\left\{P^{r}\left(\omega \right)\right\}}{1-exp\left[\left(\hbar \omega -\mu \right)/\left(K_{B}T\right)\right]}$$

together with the auxiliary variable: $\Lambda \left(\vec{k},\omega \right)\frac{P^{r}(\vec{k},\omega )}{1-exp\left[\left(\hbar \omega -\mu \right)/\left(K_{B}T\right)\right]}$, leading to the relation:

(10) $$P^{<}(\vec{k},\omega )=-2iIm\{\Lambda (\vec{k},\omega )\}$$

Expressing $P^{r}(\vec{k},\omega )$ from Equation (8) in terms of $\Lambda \left(\vec{k},\omega \right)$ the corresponding integro-differential equation becomes

(11) $$\left(\hbar \omega -\Delta e\left(\vec{k}\right)+i\Gamma \right)\Lambda \left(\vec{k},\omega \right)+\vartheta \underset{\vec{k}\prime }{\sum }W_{\vec{k}-\vec{k}\prime }\Lambda \left(\vec{k}\prime ,\omega \right)=-ℬ$$

where $ℬ=-\frac{\tanh\left[\left(\hbar \omega -\mu \right)/\left(2K_{B}T\right)\right]}{1-exp\left[\left(\hbar \omega -\mu \right)/\left(K_{B}T\right)\right]}=\frac{1}{1+exp\left[\left(\hbar \omega -\mu \right)/\left(K_{B}T\right)\right]}$.

**Figure 3:**
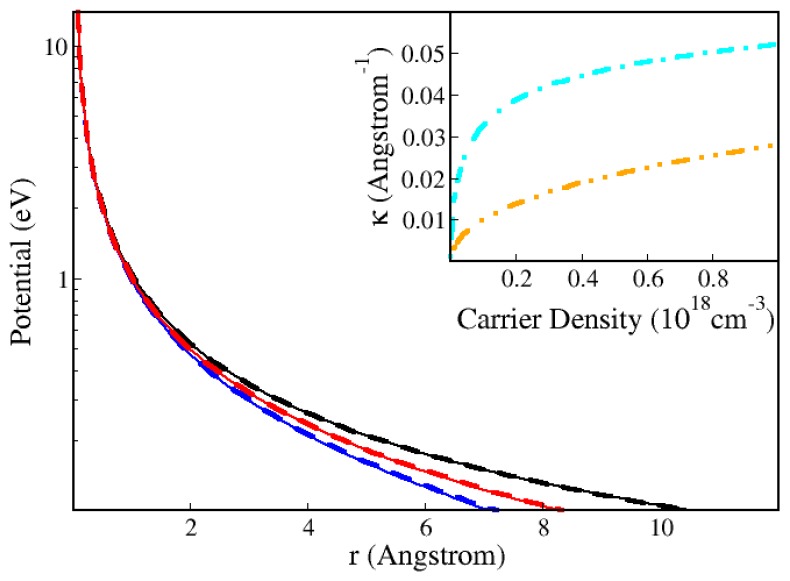
Comparison of the Hulthén (dashed lines) and Yukawa (thin solid lines) potentials as a function of distance. The black curves are for *T* = 10 K and *N* = 10^10^ carriers/cm^3^. The blue curves are for *T* = 10 K and *N* = 10^18^ carriers/cm^3^ and the red curves are for *T* = 300 K and *N* = 10^18^ carriers/cm^3^. Both cases depend on the temperature *T* and carrier density *N* through the inverse screening length κ and the inset explains the results, because κ increases with increasing carrier density and with decreasing temperature. The dot-dashed (cyan) curve is for *T* = 10 K, while the double-dot-dashed (orange) curve is for *T* = 300 K.

Before proceeding, the Hulthén potential [21,22,23,24] should be revised. The usual approximation for a static 3D screened potential is the Yukawa potential, $W_{Y}\left(\left|\vec{r}\right|\right)=-e^{2}\exp\left(-\kappa \left|\vec{r}\right|\right)/\left(\epsilon_{0}r\right)$. However, the corresponding Schrödinger equation does not have known analytical solutions. In contrast, the Hulthén potential: $W\left(\left|\vec{r}\right|\right)=-2e^{2}\kappa \epsilon_{0}^{-1}/\left(\left(\exp(2\kappa \left|\vec{r}\right|\right)-1\right))$, has known analytical solutions that have proven to be very useful for the description of bulk absorption [22]. Recent applications have confirmed its relevance to explain experimental luminescence studies [7,8,9]. Figure 3 shows that, in the range of carrier densities and temperatures of interest, the Yukawa potential can be replaced by the Hulthén potential with negligible differences in numerical values.

The Fourier transform of the Hulthén potential has an analytical expression,

(12) $$W_{\vec{q}}=-\frac{2\pi e^{2}}{\Omega \epsilon_{0}^{\prime }\kappa \left|\vec{q}\right|}Im\left\{\psi^{\prime }\left(1+\frac{i\left|\vec{q}\right|}{2\kappa }\right)\right\}$$

where Ω is the sample volume, $\psi^{\prime }$ is the Trigamma function [26], κ is the screening wavenumber and by including ϑ in W at Equation (11), $\epsilon_{0}^{\prime }=\epsilon_{0}/\vartheta$. Analytical approximations for μ and κ are given in Ref. [7]. Note that the bandgap renormalization including Coulomb hole and screened exchange corrections reads

(13) $$\Delta E_{g}=-\frac{e^{2}\kappa }{\epsilon_{0}^{\prime }}-\underset{\vec{q}}{\sum }W_{\vec{q}}\left(f_{e}\left(\vec{q}\right)+f_{h}\left(\vec{q}\right)\right).$$

**Figure 4:**
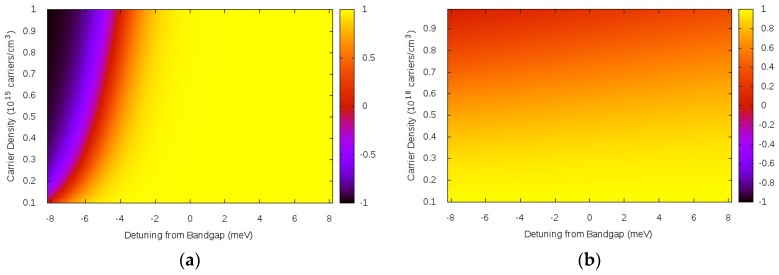
Inversion factor, ϑ≡tanh[β(ℏω−μ)/2] for GaAs as function of $\mathrm{Detuning}\;\mathrm{from}\;\mathrm{Bangdap}=\hbar \omega -E_{g}$ and ranges of densities consistent with low temperature luminescence, (**a**) *T* = 10 K [7,8,9] and (**b**) high temperature nonlinear absorption *T* = 300 K [14,15,16,22].

The Fermi functions $f_{e},\;f_{h}$ are evaluated at the peak of the spectral function for each particle, i.e., in Equation (5), $\hbar \omega =e_{\lambda }\left(\vec{k}\right)$. More details are given in Ref. [7]. Equation (13) goes beyond phenomenological term for the bandgap shift [21,22,23], and also, in contrast to those, here we can in principle take into account a reduction in the Coulomb interaction due to phase space filling through the factor ϑ. Note however that in the range of carrier densities and temperatures of interest ϑ≈1 i.e., $\epsilon_{0}^{\prime }\approx \epsilon_{0}$, as shown in Figure 4.

At quasi-equilibrium, used in Refs. [7,8,9,14,15,16,22], the total chemical potential μ is calculated self-consistently with the many body renormalization of the bandgap $E_{g}$ and can be written exactly as $\mu =E_{g}+\tilde{\mu }$, where $\tilde{\mu }$ is the total free carrier chemical potential calculated from the bottom of each band. In other words, the inversion factor can be equivalently written as $\vartheta \equiv \tanh\left[\beta \left(\hbar \omega -E_{g}-\tilde{\mu }\right)/2\right]$, and it is now clear why Figure 4 has the detuning $\hbar \omega -E_{g}$ in the *x*-axis. Furthermore, the 3D exciton binding energy for GaAs is 4.2 meV and there is no luminescence of absorption for a detuning below 4.2 meV, unless there are deep levels due to impurities and defects, which are not considered here. Thus the approximation, ϑ≈1 i.e., $\epsilon_{0}^{\prime }\;\approx \epsilon_{0}$ for the dielectric constant used in the Hulthén potential is clearly excellent in the low power luminescence case. Nonlinear absorption studies are only meaningful away from population inversion leading to optical gain, i.e., the studies are in the range ϑ≥1. Thus a decreasing occupation reflects phase space feeling and even for $\epsilon_{0}^{\prime }\neq \epsilon_{0}$ the approach is valid. In order to study the gain regime, the approximation used in the literature is to make at the Hulthén potential $\epsilon_{0}^{\prime }=\epsilon_{0}$ and consider the inversion factor only on the right hand side of Equation (8). In other words, in the traditional “plasma theories” for bulk semiconductor absorption and gain, phase space filling (ϑ≠1) is not taken into account. Since in the high density case where gain develops, the Hulthén potential decreases due to screening, which described by large κ in Equation (12), there is still good agreement with experiments. See e.g., Refs. [22,23].

Equation (11) can now be Fourier-transformed

(14) $$f\left(\vec{r}\right)=\frac{\Omega }{{\left(2\pi \right)}^{3}}\int^{\text{​}}f_{\vec{q}}e^{-i\vec{q}\cdot \vec{r}}d^{3}q,\;f_{\vec{q}}=\frac{1}{\Omega }\int^{\text{​}}f\left(\vec{r}\right)e^{i\vec{q}\cdot \vec{r}}d^{3}r,$$

(15) $$\left[\hbar \omega +i\Gamma -E_{g}+\frac{\hbar^{2}\nabla^{2}}{2m^{*}}+W\left(\vec{r}\right)\right]\Lambda \left(\vec{r},\omega \right)=-\Omega ℬ\delta \left(\vec{r}\right).$$

Here Ω is the sample volume, $\delta \left(\vec{r}\right)$ denotes the Dirac delta function. Expanding $\Lambda \left(\vec{r},\omega \right)$ in the basis of eigenstates of the Hamiltonian: $ℋ=-\frac{\hbar^{2}\nabla^{2}}{2m_{r}}-W\left(\vec{r}\right)$,

(16) $$-\left[\frac{\hbar^{2}\nabla^{2}}{2m_{r}}+W\left(\vec{r}\right)\right]\psi_{\nu }\left(\vec{r}\right)=E_{\nu }\psi_{\nu }\left(\vec{r}\right),\;\Lambda \left(\vec{r},\omega \right)=\underset{\mu }{\sum }a_{\mu }\left(\omega \right)\psi_{\mu }\left(\vec{r}\right).$$

Thus, Equation (15) can be rewritten as

(17) $$\left[\hbar \omega +i\Gamma -E_{g}-ℋ\right]\underset{\mu }{\sum }a_{\mu }\psi_{\mu }=-\Omega ℬ\delta \left(\vec{r}\right).$$

Projection onto state ν yields

(18) $$a_{\nu }\;\left(\hbar \omega +i\Gamma -E_{g}-E_{\nu }\right)=-ℬ\;\Omega \;\psi_{\upsilon }^{*}\left(0\right).$$

Substitution into Equation (16)

(19) $$\Lambda \left(\vec{r},\omega \right)=\;\underset{\nu }{\sum }-\frac{\Omega ℬ\psi_{\nu }^{*}\left(0\right)}{\hbar \omega -E_{g}-E_{\nu }+i\Gamma }\psi_{\nu }\left(\vec{r}\right).$$

Fourier-transforming back to *k*-space

(20) $$\Lambda \left(\vec{k},\omega \right)=\underset{\nu }{\sum }\frac{-ℬ\psi_{\nu }^{*}\left(0\right)}{\hbar \omega -E_{g}-E_{\nu }+i\Gamma }\int^{\text{​}}d^{3}r\;\psi_{\nu }\left(\vec{r}\right)e^{i\vec{k}\cdot \vec{r}}.$$

From $\Lambda \left(\omega \right)=\sum_{\vec{k}}\Lambda \left(\vec{k},\omega \right)$ and $\sum_{\vec{k}}e^{i\vec{k}\cdot \left(\vec{r}-\vec{r}\prime \right)}=\Omega \;\delta \left(\vec{r}-\vec{r}\prime \right)$, a closed expression can be obtained.

(21) $$\Lambda \left(\omega \right)=2\underset{\nu }{\sum }\frac{-\Omega ℬ{\left|\psi_{\nu }\left(0\right)\right|}^{2}}{\hbar \omega -E_{g}-E_{\nu }+i\Gamma }$$

where a factor 2 for spin has been explicitly written out of the summation over all quantum numbers. Introducing and combining Equations (1), (2), (10) and (21) leads to

(22) $$P^{<}\left(\omega \right)=-i\frac{8\pi e^{2}}{\Omega c^{2}}{\left|\Pi \right|}^{2}Im\left\{\Lambda \left(\omega \right)\right\}=\frac{16\pi^{2}e^{2}}{c^{2}}ℬ{\left|\Pi \right|}^{2}\underset{\nu }{\sum }\delta_{\Gamma }\left(\hbar \omega -E_{g}-E_{\nu }\right)\;{\left|\psi_{\nu }\left(0\right)\right|}^{2},$$

(23) $$I\left(\omega \right)=\frac{4\hbar \omega^{2}e^{2}\;{\left|\Pi \right|}^{2}}{c^{3}\left(1+exp\left[\beta \left(\hbar \omega -\mu \right)\right]\right)}\underset{\nu }{\sum }{\left|\psi_{\nu }\left(0\right)\right|}^{2}\delta_{\Gamma }\left(\hbar \omega -E_{g}-E_{\nu }\right),$$

where $\delta_{\Gamma }=\frac{1}{\pi }\frac{\Gamma }{{\left(\hbar \omega -E_{g}-E_{\nu }\right)}^{2}+\Gamma^{2}}$ reduces to a Dirac delta function for Γ→0. The velocity matrix element is expressed in terms of the dipole moment matrix element and the fundamental bandgap as $\left|\Pi \right|=\left(E_{g}^{0}/\hbar \right)\left|S\left|x\right|X\right|$. Next, the Schrödinger Equation for the Hulthén potential must be solved, so that $\psi_{\nu }\left(0\right)$ can be inserted in Equation (23). The first step is to separate the wavefunction in radial and angular parts. The label ν thus spans the set {n,l,m},

(24) $$\psi_{\nu }\left(\vec{r}\right)=f_{nl}\left(r\right)Y_{lm}\left(\theta ,\varphi \right)$$

The corresponding Schrödinger Equation, which is a generalized Wannier equation [23] can be cast in the form:

(25) $$\left[-\frac{\hbar^{2}}{2\mu }\frac{1}{r^{2}}\left(r\frac{\partial }{\partial r}\right)\left(r\frac{\partial }{\partial r}\right)+\frac{1}{r}\frac{\partial }{\partial r}-\frac{L^{2}}{\hbar^{2}r^{2}}\right]\psi_{\nu }\left(\vec{r}\right)-\frac{e^{2}\kappa }{\varepsilon_{o}a_{0}^{2}\left(\exp(2\kappa r\right)-1)}\psi_{\nu }\left(\vec{r}\right)=E_{\nu }\psi_{\nu }\left(\vec{r}\right)$$

The energy eigenvalues depend only on the {n,l} quantum numbers, and thus we can replace $E_{n}$ by $E_{nl}$. Introducing the 3D Rydberg $e_{0}$ and Bohr Radius $a_{0}$, as well λ=2κ, $g=1/\left(\kappa a_{0}\right)$ and $\epsilon_{nl}=E_{nl}/\left(\varepsilon_{0}a_{0}^{2}\right)$, leads to

(26) $$-\frac{d^{2}f_{nl}}{dr^{2}}-\frac{2}{r}\frac{df_{nl}}{dr}+\left[\frac{l\left(l+1\right)}{r^{2}}-\frac{g\lambda^{2}}{e^{\lambda r}-1}\right]f_{nl}=\epsilon_{nl}f_{nl}$$

Note that the angular momentum operator has been applied to the wavefunction directly from Equation (25) to Equation (26), i.e., $L^{2}\psi_{\nu }\left(\vec{r}\right)=l\left(l+1\right)\hbar^{2}\psi_{\nu }\left(\vec{r}\right).$ Only solutions that do not vanish at r=0 contribute to the emitted power, so l=0 is selected. The labels “nl” will be dropped at the moment to simplify the notation,

(27) $$-\frac{d^{2}f}{dr^{2}}-\frac{2}{r}\frac{df}{dr}-\frac{g\lambda^{2}}{e^{\lambda r}-1}f=\epsilon f.$$

Introducing u=rf, and β=−ϵλ2, Equation (27) is transformed into

(28) $$\frac{d^{2}u}{dr^{2}}+\frac{g\lambda^{2}}{e^{\lambda r}-1}u=\beta^{2}\lambda^{2}u.$$

The auxiliary variables, $z=1-e^{-\lambda r}$ and $w=\frac{u}{z{\left(1-z\right)}^{\beta }}$, lead to the equation

(29) $$z\left(z-1\right)\frac{d^{2}w}{dz^{2}}+\left[2-z\left(2\beta +3\right)\right]\frac{dw}{dz}-w\left[2\beta +1-g\right]=0$$

which reduces to the Hypergeometric Equation [26,27],

(30) $$z\left(z-1\right)\frac{d^{2}F}{dz^{2}}+\left[\eta -z\left(\xi +\zeta +1\right)\right]\frac{dF}{dz}-\xi \;\zeta \;F=0$$

(31) $$\xi =\beta +1+\sqrt{g+\beta^{2}},\;\zeta =\beta +1-\sqrt{g+\beta^{2}},\;\eta =2$$

(32) $$F\left(\xi ,\zeta ,\eta ;z\right)=1+\frac{\xi \zeta }{\eta }\frac{z}{1!}+\frac{\xi \left(\xi +1\right)\zeta \left(\zeta +1\right)}{\eta \left(\eta +1\right)}\frac{z^{2}}{2!}+\ldots$$

The generalized Wannier Equation, Equation (25), has two types of solutions: bound states for $\epsilon_{\nu }<0$ and unbound solutions for $\epsilon_{\nu }>0$. The wavefunctions and eigenvalues are thus different and it makes sense to study each case separately and then add all contributions when a sum over all possible ν as required from Equation (23).

### 2.2. Bound States

For bound states, $\epsilon_{\nu }<0$, and thus β is real. The function f must be normalized, so $\underset{r\to \infty }{\lim}f\left(r\right)=0$. This is achieved for either ξ or ζ a negative integer. Set e.g., ξ=1−n, n=1,2,3, …, in Equation (31), leading to

(33) $$\beta \equiv \beta_{n}=\frac{1}{2n}\left(g-n^{2}\right),\;\epsilon_{n}=-\lambda^{2}\beta_{n}^{2}$$

Thus, this selected solution of the Hypergeometric equation that does not vanish at r=0 is

(34) $$u\left(r\right)=Nz{\left(1-z\right)}^{\beta }F(1-n,1+g/n,2;z),\;$$

where the normalization factor N is defined by the normalization condition:

(35) $$\int_{0}^{\infty }r^{2}{\left|f\left(r\right)\right|}^{2}=\int_{0}^{\infty }{\left|u\left(r\right)\right|}^{2}=1,$$

Since the wavefunction $Y_{lm}\left(\theta ,\varphi \right)$ is normalized to 1 over angular integration. Setting 1−n≡m or equivalently n=m+1, the other relations become: 2β=g/n−n or g/n+1=2β+m+1+ν, ν=1. Equation (8.962.1) of Ref. [27] can be used leading to

(36) $$F\left(-m,2\beta +m+\nu ,\nu +1;z\right)=P_{m}^{\left(1,2\beta \right)}\left(1-2z\right)m!\Gamma \left(2\right)/\Gamma \left(m+2\right)$$

where the Legendre Polynomial $P_{m}^{\left(\alpha ,\beta \right)}$ and the Gamma function Γ have been used. Introducing x=1−2z, the normalization constant thus reads

(37) $$\begin{array}{cc} N^{-2} & =\int_{0}^{\infty }z^{2}{\left(1-z\right)}^{2\beta }{\left(\frac{m!}{\Gamma \left(m+2\right)}\right)}^{2}{\left[P_{m}^{\left(1,2\beta \right)}\left(1-2z\right)\right]}^{2}dr\\ & =\frac{1}{\lambda {\left(m+1\right)}^{2}}\int_{0}^{\infty }z^{2}{\left(1-z\right)}^{2\beta }{\left(\frac{m!}{\Gamma \left(m+2\right)}\right)}^{2}{\left[P_{m}^{\left(1,2\beta \right)}\left(1-2z\right)\right]}^{2}dz\\ & =\frac{1}{2\lambda {\left(m+1\right)}^{2}}\int_{-1}^{1}{\left(\frac{1-x}{2}\right)}^{2}{\left(\frac{1+x}{2}\right)}^{2\beta -1}{\left[P_{m}^{\left(1,2\beta \right)}\left(x\right)\right]}^{2}dx. \end{array}$$

However, $P_{m}^{\left(1,2\beta \right)}\left(-x\right)=P_{m}^{\left(2\beta ,1\right)}\left(x\right)$, see Equation (8.960) of Ref. [27] leading to

(38) $$N^{-2}=\frac{1}{2\lambda {\left(m+1\right)}^{2}}\int_{-1}^{1}{\left(\frac{1+x}{2}\right)}^{2}{\left(\frac{1-x}{2}\right)}^{2\beta -1}{\left[P_{m}^{\left(2\beta ,1\right)}\left(x\right)\right]}^{2}dx,$$

(39) $$N^{-2}=\frac{1}{2\lambda {\left(m+1\right)}^{2}}\;{\left(\frac{1}{2}\right)}^{2\beta }\left\{\int_{-1}^{1}\left(1+x\right){\left(1-x\right)}^{2\beta -1}{\left[P_{m}^{\left(2\beta ,1\right)}\left(x\right)\right]}^{2}-\left(\frac{1}{2}\right)\int_{-1}^{1}\left(1+x\right){\left(1-x\right)}^{2\beta }{\left[P_{m}^{\left(2\beta ,1\right)}\left(x\right)\right]}^{2}\right\}$$

(40) $$N^{-2}\equiv \frac{1}{2\lambda {\left(m+1\right)}^{2}}\;{\left(\frac{1}{2}\right)}^{2\beta }\left\{I_{1}-\frac{1}{2}I_{2}\right\}.$$

The solutions for the integrals above are given by Equations (7.391.1) and (7.391.5) of Ref. [27],

(41) $$I_{1}=2^{2\beta +1}\frac{\Gamma \left(2\beta +m+1\right)\Gamma \left(2+m\right)}{m!\left(2\beta \right)\Gamma \left(2\beta +m+2\right)},\;I_{2}=2^{2\beta +2\;}\frac{\Gamma \left(2\beta +m+1\right)\Gamma \left(2+n\right)}{m!\left(2\beta +2m+2\right)\Gamma \left(2\beta +m+2\right)}$$

Which combined with Equation (6.1.15) of Ref. [26], yields

(42) $$N^{-2}=\frac{2}{4\lambda \beta \left(2\beta +n\right)\left(\beta +n\right)}$$

However, $2\beta =g/n-n,\;n+2\beta =g/n,\;2n+2\beta =g/n+n,\;\beta \equiv \beta_{n}.$ Consequently,

(43) $$N^{2}=\frac{\lambda g^{3}}{2n}\left(\frac{1}{n^{2}}-\frac{n^{2}}{g^{2}}\right)$$

The intensity spectrum requires ${\left|\psi_{n}\left(0\right)\right|}^{2}$, where $\psi_{n}\left(\vec{r}\right)=NY_{00}z{\left(1-z\right)}^{\beta_{n}}F\left(1-n,1+g,2;z\right)$. Note that $Y_{00}=\frac{1}{\sqrt{4\pi }}$,

(44) $$\underset{r\to 0}{\lim}{\left|\psi_{n}\left(\vec{r}\right)\right|}^{2}=\frac{N^{2}}{4\pi }{\left(\frac{\lambda r}{r}\right)}^{2}{\left(F\left(1-n,1+g,2;0\right)\right)}^{2}=\frac{N^{2}\lambda^{2}}{4\pi }$$

Note that $\lambda^{3}=8\kappa^{3}$ and $g^{3\;}=\frac{1}{a_{0}^{3}\kappa^{3}}$. Furthermore, the bound state energies are $E_{n}=e_{0}a_{0}^{2}\epsilon =-e_{0}a_{0}^{2}\lambda^{2}\beta_{n}^{2}.$ Thus, in summary

(45) $${\left|\psi_{n}\left(0\right)\right|}^{2}=\frac{1}{\pi a_{0}^{3}}\frac{1}{n}\left(\frac{1}{n^{2}}-\frac{n^{2}}{g^{2}}\right),\;E_{n}=-e_{0}\frac{1}{n^{2}}{\left(1-\frac{n^{2}}{g}\right)}^{2}.$$

Note that ${\left|\psi_{n}\left(0\right)\right|}^{2}\geq 0\to 1/n^{2}-n^{2}/g^{2}\geq 0\to n\leq \sqrt{g}\to n\in \left\{0,Int\left\{\sqrt{g}\;\right\}\right\}$. In other words, the number of bound states that contribute to the power spectrum are determined by the size of the inverse screening length. For zero screening, g→∞.

### 2.3. Continuum States

The unbound solutions that make a continuum have positive eigenvalues, $\epsilon_{\nu }>0$, and thus imaginary $\beta_{\nu }=i\sqrt{\epsilon_{\nu }}/\lambda$. Dropping labels to simplify the development in the next equations yields

(46) $$u=Nz{\left(1-z\right)}^{i\beta }F\left(1+i\beta +\sqrt{g-\beta^{2}},1+i\beta -\sqrt{g-\beta^{2}},2;z\right),$$

which can be written for simplicity as

(47) $$F\left(a,b,c;z\right),\;a=1+i\beta +\sqrt{g-\beta^{2}},\;b=1+i\beta -\sqrt{g-\beta^{2}},c=2$$

Note that the transformation $z=1-e^{-\lambda r}$ is being used. The solution that will be later inserted in Equation (23), will be normalized in a sphere of radius r=R and asymptotic solutions, obtained a large radius R will be investigated. Next, Equation (15.3.6) from Ref. [26] is used, i.e., $F\left(a,b,c;z\right)=\frac{\Gamma \left(c\right)\Gamma \left(c-a-b\right)}{\Gamma \left(c-a\right)\Gamma \left(c-b\right)}F\left(a,b,a+b-c+1,1-z\right)+{\left(1-z\right)}^{c-a-b}\frac{\Gamma \left(c\right)\Gamma \left(c-a-b\right)}{\Gamma \left(a\right)\Gamma \left(b\right)}F\left(c-a,c-b,c-a-1+1,1-z\right).$

Furthermore, note that $\underset{R\to \infty }{\lim}z=1$ and for all values of ξ,λ,δ, F(ξ,λ,δ;0)=1. Thus,

(48) $$\underset{R\to \infty }{\lim}F\left(a,b,c;z\right)=\frac{\Gamma \left(c\right)\Gamma \left(c-a-b\right)}{\Gamma \left(c-a\right)\Gamma \left(c-b\right)}+{\left(1-z\right)}^{c-a-b}\frac{\Gamma \left(c\right)\Gamma \left(a+b-c\right)}{\Gamma \left(a\right)\Gamma \left(b\right)}$$

$\Gamma \left(c\right)=\Gamma \left(2\right)=1,\;c-a-b=-2i\left|\beta \right|,\;a+b-c=2i\left|\beta \right|,\;c-a=1-i\beta -\sqrt{g-\beta^{2}}=b^{*},\;c-b=1-i\beta +\sqrt{g-\beta^{2}}=a^{*}$, which, combined with $\Gamma \left(z^{*}\right)={\left[\Gamma \left(z\right)\right]}^{*}$ (Equation (6.1.2.3) of Ref. [26]), gives in the asymptotic limit

(49) $$F\left(a,b,c;z\right)=\frac{\Gamma \left(c-a-b\right)}{\Gamma \left(b^{*}\right)\Gamma \left(a^{*}\right)}+{\left(1-z\right)}^{-2i\beta }\frac{\Gamma^{*}\left(c-a-b\right)}{\Gamma \left(a\right)\Gamma \left(b\right)}$$

Leading to asymptotic forms of $u,\;{\left|u\right|}^{2}$

(50) $$u=N{\left(1-z\right)}^{i\lambda \beta R}\left\{\frac{\Gamma \left(c-a-b\right)}{\Gamma \left(b^{*}\right)\Gamma \left(a^{*}\right)}+\frac{\Gamma^{*}\left(c-a-b\right)}{\Gamma \left(a\right)\Gamma \left(b\right)}e^{-2i\lambda \beta R}\right\}$$

(51) $${\left|u\right|}^{2}=N^{2}{\left|\frac{\Gamma \left(c-a-b\right)}{\Gamma \left(b^{*}\right)\Gamma \left(a^{*}\right)}e^{-i\lambda \beta R}+\frac{\Gamma^{*}\left(c-a-b\right)}{\Gamma \left(a\right)\Gamma \left(b\right)}e^{i\lambda \beta R}\right|}^{2}=4N^{2}{\left|\frac{\Gamma \left(c-a-b\right)}{\Gamma \left(c-a\right)\Gamma \left(c-b\right)}\right|}^{2}{\left(\cos\left(\lambda \beta R+\xi \right)\right)}^{2}$$

where $\xi =arg\left\{\frac{\Gamma \left(c-a-b\right)}{\Gamma \left(c-a\right)\Gamma \left(c-b\right)}\right\}$. The normalization constant is thus given by $N^{-2}=\underset{R\to \infty }{\lim}4{\left|\frac{\Gamma \left(c-a-b\right)}{\Gamma \left(c-a\right)\Gamma \left(c-b\right)}\right|}^{2}\int_{0}^{R}{\left(\cos\left(\lambda \beta r+\xi \right)\right)}^{2}dr$,

(52) $$N=\sqrt{\frac{1}{2R}{\left|\frac{\Gamma \left(c-a-b\right)}{\Gamma \left(c-a\right)\Gamma \left(c-b\right)}\right|}^{2}}.$$

However, ${\left|\Gamma (-2i\left|\beta \right|\right|}^{2}=\frac{\pi }{2\left|\beta \right|\sinh\left(2\pi \left|\beta \right|\right)},$ see e.g., Equations (6.1.29) and (6.1.31) of Ref. [26], plus a little algebra deliver the continuum normalization constant

(53) $$N=\sqrt{\frac{2}{R}\frac{\pi \left|\beta \right|g\sinh\left(2\pi \left|\beta \right|\right)}{\cosh\left(2\pi \left|\beta \right|\right)-\cos\left(\sqrt{g-{\left|\beta \right|}^{2}}\right)}}.$$

The required value of the wave function at the origin can thus be expressed as

(54) $$\psi \left(0\right)=\underset{r\to 0}{\lim}N\frac{1}{\sqrt{4\pi }}\frac{z{\left(1-z\right)}^{i\left|\beta \right|}}{r}F\left(a,b,c;0\right).$$

Next, note that $\underset{r\to 0}{\lim}z=0$, but $\underset{r\to 0}{\lim}\frac{z}{r}=\lambda$, leading to

(55) $${\left|\psi \left(0\right)\right|}^{2}=\frac{\lambda^{2}}{4\pi }\frac{4\pi }{R}\frac{\left|\beta \right|g\sinh\left(2\pi \left|\beta \right|\right)}{\cosh\left(2\pi \left|\beta \right|\right)-\cos\left(\sqrt{g-{\left|\beta \right|}^{2}}\right)}.$$

The sum of continuum states becomes an integral, $\underset{\nu }{\sum }\ldots =\frac{R\lambda }{\pi }\int_{0}^{\infty }\ldots$ Introducing 2 for spin and changing variables, the continuum contribution becomes

(56) $$\underset{\nu }{\sum }2{\left|\psi_{\nu }\left(0\right)\right|}^{2}=\frac{1}{\pi a_{0}^{3}}\int_{0}^{\infty }\frac{\sinh\left(\pi g\sqrt{x}\right)}{\cosh\left(\pi g\sqrt{x}\right)-\cos\left(\pi \sqrt{4g-g^{2}x}\right)}$$

which combines with the bound states

(57) $$\underset{n}{\sum }2{\left|\psi_{n}\left(0\right)\right|}^{2}\underset{n}{\sum }\frac{2}{\pi a_{0}^{3}}\frac{1}{n}\left(\frac{1}{n^{2}}-\frac{n^{2}}{g^{2}}\right)$$

to deliver the power spectrum

(58) $$I\left(\omega \right)=\frac{I_{0}}{1+exp\left(\beta \left[\hbar \omega -\mu \right]\right)}\times \left\{\sum_{n=1}^{Int\left\{\sqrt{g}\right\}}\frac{4\pi }{n}\left(\frac{1}{n^{2}}-\frac{n^{2}}{g^{2}}\right)\delta_{\Gamma }\left(\zeta -e_{n}\right)+\int_{0}^{\infty }\frac{2\pi \;\sin h\left(\pi g\sqrt{x}\right)\;}{\cosh\left(\pi g\sqrt{x}\right)-\cos\left(\pi \sqrt{4g-g^{2}x}\right)}\delta_{\Gamma }\left(\zeta -x\right)dx\right\}.$$

Here, $\zeta =\frac{\hbar \omega -E_{g}}{e_{0}}$, $e_{n}=-\frac{1}{n^{2}}{\left(1-\frac{n^{2}}{g}\right)}^{2}$, $I_{0}=\frac{\hbar e^{2}\omega^{2}{\left|\Pi \right|}^{2}}{\pi e_{0}c^{3}a_{0}^{3}}$ and the square of the velocity matrix element is ${\left|\Pi \right|}^{2}={\left(E_{g}^{0}/\hbar \right)}^{2}{\left|S\left|x\right|X\right|}^{2}=\;\frac{E_{g}^{0}\left(E_{g}^{0}+\Delta \right)}{2m_{c}\left(E_{g}^{0}+2\Delta /3\right)}$, where the spin orbit shift, the free-carrier bandgap, and renormalized bandgap are given by $\Delta ,\;E_{g}^{0}$, and $E_{g}$.

Note that this approach does not include cavity effects, which can be introduced in the Photon Green’s functions solution following Ref. [19]. Quasi-periodic structures can also be addressed by a Green’s functions formalism as shown in Ref. [28].

## 3. Numerical Application

The goal of this section is to illustrate the approach and the many quantities and parameters used making reference to published material, where the equations are used delivering very good agreement with experimental data. Photoluminescence is a very powerful tool to characterize semiconductor materials and map specific characteristics of new devices. Equation (58) is the reference, since it delivers the emission spectrum, which can be directly compared with experimental data. The carriers generated by the photo excitation process modify the spectrum and these modifications are described in Equation (58) approximately by the corrections induced by the (screened) potential, bandgap renormalization, and changes in linewidth governed by the dephasing or scattering Γ. The temperature *T* can be measured and used as an input parameter. The carrier density *N* can be estimated by measuring the input power and its spot size when focused on the sample, but in our recent investigations, where this theory has been very successfully compared with experiments [7,8,9], it has been treated as a free parameter, which has been globally adjusted. The other parameters that characterize the material, i.e., the fundamental band gap $E_{g}^{0}$, the electron and hole effective masses $m_{e\;},\;m_{h}$, the static dielectric constant $\epsilon_{0}$, and the spin-orbit shift Δ, can either be found in the literature or robust numerical methods such as simulated annealing can be used to determine these parameters by direct comparison with experiments.

As a reference for the material parameters recently used for dilute nitrides and bismides and the corresponding bandstructure calculations that lead to the material parameters, see: Ref. [7] for GaAs_1−*x*_Bi*_x_*; Ref. [9] for InAs_1−*x*_N*_x_* and Ref. [8] for more complex quaternary materials, such as InAs_1−*x*−*y*_N*_x_*Sb*_y_*. The “s-shape” in the luminescence profiles as a function of temperature for these materials have been well explained in Refs. [7,8,9]. However, in the case of completely new materials, expecting to have general characteristics as the ternary or quaternary above, the parameters leading to the bandstructure may be unknown. This theory can be used as a numerical characterization tool as follows.

The corresponding bandstructure can depend on a number of unknown parameters for new compounds, but the approach used in Refs. [7,8,9] can be extended in the following way to extract these parameters by a systematic comparison between theory and experiments. For fixed excitation power, the luminescence can be measured for a number of different temperature points. The dephasing corresponding to different excitation processes can be calculated or taken also as a parameters. Thus, at each Temperature *T*, there is an ensemble of parameters, such as

(59) $$E=\left\{m_{e},\;m_{h},\;\Gamma ,N,E_{g}^{0}\right\}.$$

Experiments provide a series of data points measured at *T* = (*T*_1_, …, *T_N_*). The calculated luminescence spectrum will be a function of *T* and will depend on the ensemble of parameters, denoted $u_{ℭ}\left(T_{i}\right).$ The least squares method leads to estimates of the parameter ensemble E by minimizing the residual between the theoretical function and the experiments. Therefore, the problem becomes

(60) $$\underset{E}{\min}\overset{N}{\underset{i=1}{\sum }}{\left(u_{ℭ}\left(T_{i}\right)-d_{i}\right)}^{2}.$$

Trust Region-Reflective (TRR) methods deliver an efficient solution for this numerical problem and Ref. [8] gives further details of their application.

**Figure 5:**
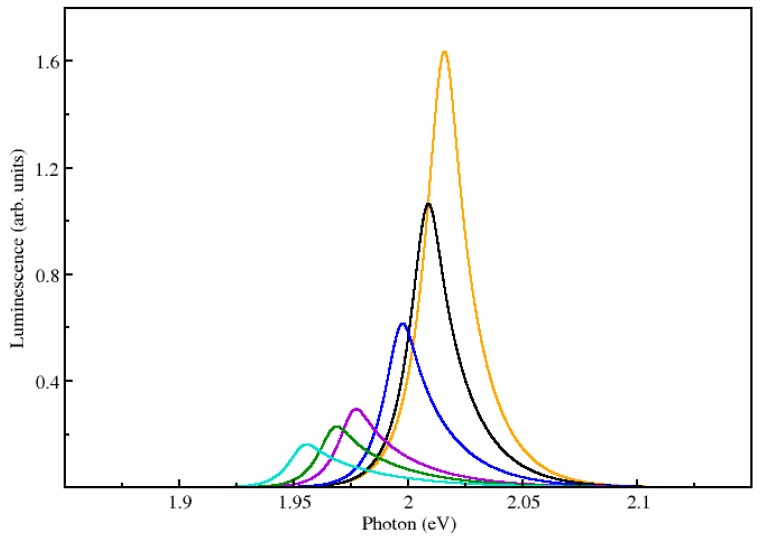
Luminescence for a GaAs–Al_0.3_Ga_0.7_As superlattice with repeated barrier and well widths equal to 2 nm. All curves have been calculated with the same broadening Γ=5.4 meV and a carrier density *N* = 10^14^ cm^−3^ From left to right, the orange, black, blue, violet, green and turquoise curves have been calculated for temperatures given respectively by *T* = 150 K, 170 K, 200 K, 250 K, 270 K, and 300 K. The corresponding free carrier bandgaps needed for input are $E_{g}^{0}$ = 2.021 eV, 2.015 eV, 2.003 eV, 1.982 eV, 1.974 eV, and 1.960 eV.

Figure 5 depicts a numerical example to further illustrate choices for the main input parameters. Short period superlattices with strong delocalization of the electron and hole wavefunctions can be described in many cases by anisotropic 3D media, characterized by in-plane and transverse (along the growth direction) effective masses and dielectric constants. The anisotropy parameter γ is given by the ratio between the in-plane $\mu_{∥}$ and perpendicular $\mu_{⊥}$ reduced effective masses, $\gamma =\mu_{∥}/\mu_{⊥}$, with $\frac{1}{\mu_{∥}}=\frac{1}{m_{e∥}}+\frac{1}{m_{h∥}}$ and $\frac{1}{\mu_{⊥}}=\frac{1}{m_{e⊥}}+\frac{1}{m_{h⊥}}$ which are calculated from the non-interacting superlattice Hamiltonian ${ℋ}_{0}$, $\frac{1}{m_{i∥}}=\hbar^{-2}\partial^{2}/\partial k_{i∥}^{2}\langle \Psi \left|{ℋ}_{0}\right|\Psi \rangle$, $\frac{1}{m_{i⊥}}=\hbar^{-2}\partial^{2}/\partial k_{i⊥}^{2}\langle \Psi \left|{ℋ}_{0}\right|\Psi \rangle$, for *i* = *e*, *h*.

These can be calculated from the corresponding free carrier Hamiltonian, and full details of the method, which has led to good agreement with experimental data, can be found in Refs. [15,16,21,29]. Figure 5 shows calculated luminescence using the modified parameters determined by anisotropic medium theory for a short period GaAs–Al_0.3_Ga_0.7_As superlattice with repeated barrier and well widths equal to 2 nm. The resulting effective masses are $m_{e∥}\approx$ 0.08; $m_{h∥}\approx$ 0.12, $m_{e⊥}\approx$ 0.08, $m_{h⊥}\approx$ 0.53. These lead the anisotropy parameter γ= 0.67. The resulting exciton binding energy and Bohr radius are given respectively by $e_{0}=5.37$ meV and $a_{0}=$ 11.04 nm.

Except of course for the actual value of the bandgap, which is larger due to quantum confinement, the strong delocalization of electrons and holes in this short period superlattice make the evolution of the luminescence with temperature look qualitatively similar to a three dimensional (bulk) semiconductor, notably the evolution of the line-shape. This is quite similar to the calculations presented in Ref. [9], which are in very good agreement with the experiments discussed in Ref. [13].

## 4. Conclusions

Photon Green’s function techniques have been used to study different types of luminescence over the years, but the solutions are typically numerically intensive and not accessible for experimentalists or non-specialists. To bridge this gap, a simple analytical solution of the relevant Green’s functions was necessary and the approach described here meets those needs. Notably, the evolution of luminescence with temperature has been successfully compared for GaAsBi [7] and InAs(N,Sb) [8] and InAsN [9] dilute semiconductors. Furthermore, the approach has been used to predict nonlinearities in short period superlattices treaded as anisotropic three-dimensional media [14,15,16]. However, details of the mathematical steps needed to achieve the final formulas used in these publications have not been previously presented and they are given in this review, complemented by numerical results demonstrating the range of validity of the main approximations used in the development of the approach. To complete the picture, an application section shows how to use the method as a numerical characterization machine, and the main parameters needed in typical simulations are illustrated with results for luminescence of short period superlattices. Screening of the Coulomb interaction between electrons and holes is discussed by means of the Hulthén potential and the steps provided are suitable as a guideline to the study of other interacting potentials of interest. They can be followed for further development of a suite of algorithms for efficient and easily programmable numerical characterization tools for a host of new bulk materials or superlattices that can described be as effective 3D media using anisotropic medium approximations.
